# The New SARS-CoV-2 Variants and Their Epidemiological Impact in Mexico

**DOI:** 10.1128/mbio.01060-21

**Published:** 2022-08-16

**Authors:** Rodrigo García-López, Estibalitz Laresgoiti-Servitje, Roselyn Lemus-Martin, Alejandro Sanchez-Flores, Carlos Sanders-Velez

**Affiliations:** a Instituto de Biotecnología, Universidad Nacional Autónoma de México, Cuernavaca, Mexico; b Clinical Medical Sciences, Faculty of Medicine and Health Sciences, Tecnologico de Monterrey, Mexico City, Mexico; c HPQC Labs, Ontario, Canada; d School of Pharmacy, University of Nottingham, Nottingham, United Kingdom; Louisiana State University Health Sciences Center Shreveport; Albert Einstein College of Medicine

**Keywords:** SARS-CoV-2 variants, genomic vigilance, vaccination, COVID-19 pandemic

## Abstract

The COVID-19 disease caused by the severe acute respiratory syndrome coronavirus 2 (SARS-CoV-2) virus started its deadly journey into a global pandemic in Wuhan, China, in December 2019, where it was first isolated. Subsequently, multiple variants of the virus have been identified worldwide. In this review, we discuss the overall landscape of the pandemic in Mexico, including its most prevalent variants, their surveillance at a genomic level, and how they impacted the epidemiology of the disease. We also evaluate the heterologous vaccination in Mexico and how it may have influenced group immunity and helped mitigate the pandemic. Finally, we present an integrated view that could help us to understand the pandemic and serve as an example of the situation in Latin America.

## INTRODUCTION

The viral family *Coronaviridae* is composed of a widespread group of enveloped RNA viruses that infect respiratory tract cells in vertebrates. Seven such viruses are associated with respiratory disease in humans. Two common cold viruses from the genus *Alphacoronavirus* (HCoV-229E, HCoV-NL63) and five from *Betacoronavirus*, severe acute respiratory syndrome coronavirus 2 (SARS-CoV), Middle East respiratory syndrome (MERS-CoV), and SARS-CoV-2, which have caused pandemics, and two common cold viruses (HCoV-OC43 and HcoV-HKU1) (1). SARS-CoV-2 presents distinct molecular features that allow it to infect other cell types apart from those in the respiratory tract. Lungs are the most affected organ since viral entry occurs preferentially via the receptor for the angiotensin-converting enzyme 2 (ACE2), which is most abundant on the surface of type II alveolar cells. The ACE2 receptor recognizes the spikes on the virion’s surface. This type of entry depends on a proteolytic cleavage, carried out by transmembrane serine protease 2 (TMPRSS2), which is also abundant in lung cells. Recently, certain variants (Omicron) have specialized in an alternative endosomal entry, where TMPRSS2 is not readily available, thus preferentially infecting cells of the nasopharyngeal tract (2–4).

Unlike other coronaviruses, SARS-CoV-2 causes a systemic disease, known as the coronavirus disease 2019 (COVID-19). The impact of its pandemic has been uneven worldwide. Latin America has been severely affected by the disease, with higher fatality rates than in western European countries. Mexico was particularly vulnerable due to underlying population risk factors such as cardiovascular diseases, including dyslipidemia and hypertension, and type 2 diabetes (5). This resulted in a syndemic situation, where poverty and limiting social factors (such as access to health care) were determinants in the epidemiological outcome.

The COVID-19 pandemic has caused an overload of health care services worldwide, with the predicted disruption of regular health services expected to last for a long time, particularly in low- and middle-income countries (6).

In this review, we aim to address some key aspects of epidemiology, vaccination, and genomic surveillance to describe the first 2 years of the COVID-19 pandemic in Mexico.

## DIAGNOSTIC TESTS

Globally, common COVID-19 symptoms include fever, cough, odynophagia, myalgia, and cephalgia, similar to influenza. Rare symptoms, such as abdominal pain, vomiting, polypnea, conjunctivitis, rash, anosmia, and others have been reported in less than 10% of patients, thus making the differential diagnosis a challenging scenario (7–10). While SARS-CoV-2 variants share most symptoms, the Omicron variant is primarily associated with upper respiratory symptoms (rhinorrhea) (11). Due to its difficult diagnosis, viral presence must be confirmed directly (12).

Testing for the presence of viral genetic material through reverse transcription-PCR (RT-PCR) is the primary gold standard diagnostic in the official Mexican surveillance. RT-PCR has a high specificity, targeting multiple SARS-CoV-2-unique genes, and sensitivity, as amplification copies low-quantity DNA (13). However, sensitivity may vary depending on the type and quality of the sample, the target genes, and the timing of sample collection (14). Lower sensitivity may be observed when samples are obtained early during infection (3 days or prior), as the virus must replicate in order to become detectable (15). Likewise, nasopharyngeal swabs have shown higher sensitivity than oropharyngeal ones (16). RT-PCR tests are also restricted by high-end technical and expertise requirements. Rapid antigen tests are also accepted for official diagnostics in Mexico. These detect the molecular structure of the spikes on the surface of the SARS-CoV-2 virion directly. Like RT-PCR, they are highly specific but have a short sensitivity window in which they can be used, spanning from early infection until it peaks, as it depends on the total number of circulating virions. These are most sensitive in symptomatic patients (72% compared to 58% asymptomatic) and in the early stages of the disease (78% in the first week of symptom onset versus 51% after the first week of symptom onset) (17). Sensitivity has been reported to drop to 62% for the Omicron variant (18). Regarding other tests, there is little evidence favoring their use in clinical settings (19).

It should be noted that both RT-PCR and antigen tests are meant only for epidemiological surveillance but not to determine SAR-CoV-2 variants, as this requires genomic surveillance, a different set of higher-specialization techniques involving full genomic sequencing, which cannot be carried out in clinics due to their prohibitive costs and technical requirements (20). Genomic surveillance consists of analyzing the whole genome contained within the virus in a sample in order to analyze all extant mutations and trace their evolution. Each variant is defined by a specific array of mutations that are used to define the exact sublineage. In Mexico, genomic surveillance is carried out by academic and public institutions.

By design, official testing has been consistently low relative to Mexico’s current population, as only symptomatic patients are systematically tested, with 14,130,791 total tests carried out officially, or else 112,136.65 tests per million people (population, 126,014,024 [21]). According to the most recent official guidelines (22), only 10% of all ambulatory patients have requested a biological sample for testing (100% in the case of pregnant patients). In contrast, all COVID-19 hospitalized patients are considered, and both antigen tests and RT-PCR are included in epidemiologic surveillance. Only a few other private, university, and military institutions have been certified to contribute toward national surveillance.

## SARS-CoV-2 EVOLUTION AND ITS ROLE IN THE PANDEMIC

Mutations are a natural result of the replication of genetic material in biological organisms. Coronaviruses have proofreading mechanisms that correct part of these mutations, resulting in a relatively low mutation rate for RNA viruses (10^−6^, while influenza has 3 × 10^−5^) (23, 24). Mutations providing evolutionary advantages at the molecular level, such as a better recognition by the target cell, improved cell entry, or immune cell evasion are frequently kept and have the chance of becoming fixated in each viral lineage. SARS-COV-2 is a single-stranded positive-sense RNA virus with a 29.9-kb linear genome (25). Structurally, SARS-CoV-2 has 12 main open reading frames (ORFs), including genes coding four main structural proteins, spike (S), nucleocapsid (N), envelope (E), and membrane (M). Also, 2 large polyproteins (ORF1ab) are further processed into 16 nonstructural proteins involved in viral replication and immunity evasion, and there are 6 other accessory proteins (ORFs 3a, 6, 7a, 7b, 8, and 9b) (26). The S protein forms the recognizable “crown of spikes” on the surface of the virion, characteristic of the coronavirus family, and is required for viral entry (27). The S protein is recognized by ACE2 receptors on the surface of the host cell, followed by cleavage by TMPRSS2 that separates its two subunits and allows the virus to fuse to its membrane and enter the cell. The S protein is highly conserved in *Betacoronavirus* species, and thus the previous research into MERS-CoV and SARS-CoV was instrumental in the development of vaccines against SARS-CoV-2, most of which have been targeted at this protein (28). Due to its important role in the viral replication cycle, the SARS-CoV-2 spike protein has accumulated a wide range of mutations, with additional selection pressure by vaccination and concentrated in the receptor-binding domain and the N-terminal domain (29). While these mutations have resulted in variants that are capable of evading the immune response, increase its transmission, and might affect the mortality rate, it must be noted that RNA viruses have a high error rate, and thus viral lineages emerge with various levels of pathogenicity.

Mutations are used as unique fingerprints for the study of SARS-CoV-2 lineages through phylogenetic analysis, enabling tracking and classification of variants and their evolution globally; most available SARS-CoV-2 whole genomes are deposited in the EpiCoV database of the Global Initiative on Sharing All Influenza Data (GISAID) (30), a standardized international collaboration including clade classification by Nextstrain (31) and lineages with the Phylogenetic Assignment of Named Global Outbreak LINeages (Pangolin) universal nomenclature classification (32). Additionally, the World Health Organization (WHO) has identified variants of concern (VOCs), variants of interest (VOIs), and variants under monitoring (VUMs), based on their impact on global public health (33), assigning Greek letters to the most relevant for the global pandemic. Their criteria consider the virus’ mutations, increased transmission rate and virulence, and decreased effectiveness of public health and social measures or available diagnostics, vaccines, or therapeutics.

In May 2020, the D614G mutation in the spike protein became prevalent in SARS-CoV-2 genomes worldwide, giving rise to lineage B (Pango nomenclature), which still dominates the phylogenetic landscape to this date. This was reported to provide enhanced infectivity, competitive fitness, viral load, and transmission in human and animal models (34, 35). As of the time of writing this article, five main VOCs have been relevant during different stages of the pandemic in Mexico: Alpha, Beta, Gamma, Delta, and Omicron, but the current landscape is dominated by the Omicron lineage, which carries over 50 mutations (more than 30 in the spike).

## GENOMIC SURVEILLANCE IN MEXICO

Keeping track of the SARS-CoV-2 variants, virtually in real time, has been possible due to the genomic surveillance that has been installed in several countries. The WHO has called on their partner countries to install genomic surveillance strategies for pathogens with pandemic or epidemic potential, such as SARS-CoV-2, for a better understanding to take public health actions (36). Using fast, reliable, and low-cost sequencing technologies, many countries have implemented SARS-CoV-2 genomic surveillance to detect which variants are being spread in their population.

Sequencing technologies have enabled large-scale genomic surveillance of SARS-CoV-2 since thousands of samples are being sequenced worldwide. Amplicon sequencing has become the most widely adopted method due to its simplicity and cost-effectiveness for SARS-CoV-2 (compared to metagenomics and whole-genome sequencing). (37, 38). Briefly, the protocol consists of the amplification of cDNA obtained from viral RNA from SARS-CoV-2-positive patients; using protocols for Illumina (39) and Nanopore (40), it is possible to reconstruct the whole viral genome from each genome-wide set of amplicons with enough sequencing depth to call the exact mutations that define it.

**FIG 1:**
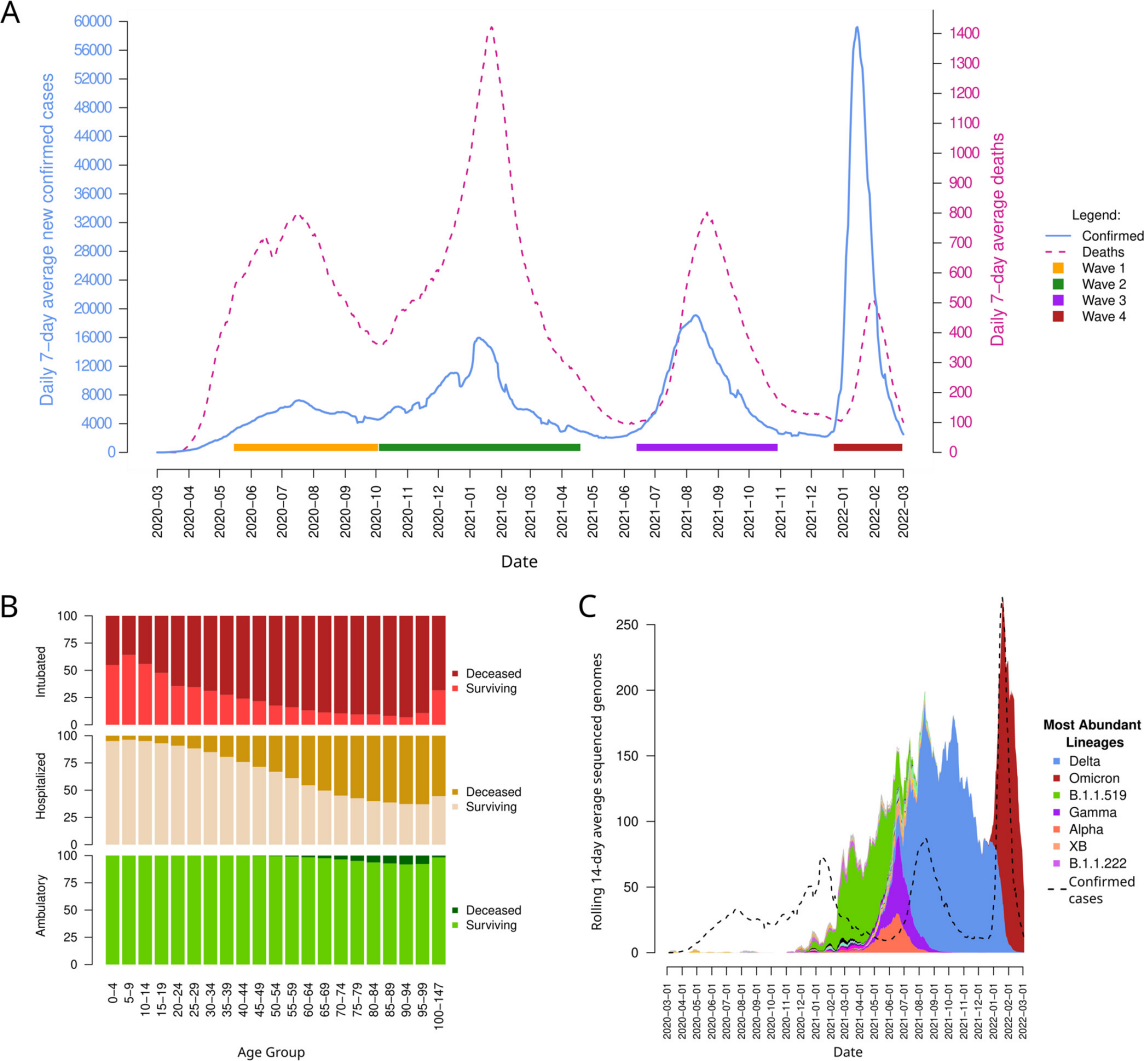
Epidemiological and genomic surveillance in Mexico. (A) Daily average of new confirmed cases and deaths in Mexico in the context of four pandemic waves. The left *y* axis portrays the number of newly confirmed patients diagnosed daily, and the right *y* axis is the total number of deaths on each date. Both metrics were calculated as rolling 7-day averages using the date when each patient was registered. The first day of each month is marked on the X-axis. (B) Percentage of deceased and surviving patients throughout the pandemic as shown by age categories (5-year step). The type of patient is shown separately. (C) Average sequenced genomes per rolling 14-day period based on the day of collection of each sample. The top 6 most abundant lineages are shown in the legend. Confirmed cases are shown in a dashed line for context only.

In Mexico, genomic surveillance was carried out by a joint effort between the governmental Instituto de Referencia Epidemiológica (InDRE; samples of epidemiological interest) and Instituto Nacional De Medicina Genomica (INMEGEN; mostly samples from Mexico City) and the academic Consorcio Mexicano de Vigilancia Genomica (CoViGen-Mex; nationwide sampling from hospitals) sequencing-capable institutions. As of 28 February 2022, 54,489 samples had been processed and deposited in GISAID. Even though these account for <1% of all confirmed cases in the country, most were sequenced after February 2021 and increased along with subsequent epidemic surges. Based on the Pangolin classification, 90 different lineages (with at least 10 observations) have been detected in Mexico, including Alpha, Beta, Gamma, Delta, and Omicron VOCs (46 if sublineages are collated). Other high-frequency variants that were detected before the onset of VOCs were lineages B.1.1.222 and its descendant B.1.1.519, which were more successful in Mexico than in the rest of the world (41, 42), and recombinant variant XB (43) (Fig. 1C). Variants B.1.1.222 and B.1.1.519 present mutations in the spike and nucleocapsid proteins, specifically, T478K, P681H, and T732A in the spike, within the B.1.1.519 lineage, which was derived from the B.1.1.222 lineage, which has been the dominant virus in Mexico from 2020 to the writing of this manuscript. The B.1.1.519 variant was found in Mexico City, and phylogeographic analysis suggests that it might have emerged around mid-September 2020 (41, 44).

Genomic surveillance contributes to high-resolution epidemiology that allows authorities to make decisions based on the distribution of the circulating variants. If the number of cases increases rapidly, it can be determined if the higher transmission is related to a certain variant, either one already reported or a new one. Furthermore, genomic surveillance allows the evaluation of vaccine effectiveness, where some variants are capable of spreading in highly vaccinated populations, such as in the case of the Omicron lineage (45).

## SARS-CoV-2 EPIDEMIOLOGY IN MEXICO

Throughout the pandemic, official figures relating to COVID-19 cases in Mexico have been made readily available to the general public by the governmental directorate on epidemiology (https://www.gob.mx/salud/documentos/datos-abiertos-152127) on a daily basis. As of 28 February 2022, a total of 15,089,355 cases had been officially registered, out of which, 8,916,487 were negative for SARS-CoV-2, while 5,508,629 were confirmed either through direct clinical testing (94.45%; Fig. 1A), by patient association (5.28%), or by review by a panel of medical experts (0.27%). Most of the remaining 664,239 cases are considered potential infections, with either no available sample, indeterminate tests, or pending results. These data represent just a sample of all cases in Mexico, and most of them have been collected in public hospitals and health institutions (94.91%), as well as a minor contribution from institutions in the private (4.17%), military (0.43%), industrial (0.44), and university (0.05%) settings. Of all confirmed cases, 51.92% were female and 48.07% were male. Most cases were detected in highly densely populated regions from the capital, Mexico City (27.94%), its neighboring State of Mexico (6.21%), and Guanajuato (4.97%) in the central region, Nuevo León (5.56%), in the north and Jalisco (4.15%) in the west. The remaining 27 states reported <4% of all cases.

As of the same date, 318,149 SARS-CoV-2-confirmed patients had died (Fig. 1A), for a 5.78% lethality (38.45% female, 61.55% male). This accounts for roughly three-fourths of the total COVID-19-related deaths estimated by the National Public Health Institute (INSP) based on official public records and revised death certificates (46). By the end of 2021, they summed a total of 461,561 cumulative COVID-related deaths since the beginning of the pandemic (calculations were based on percentiles from the previous 5 years). These excess deaths include belatedly diagnosed patients that died, those that failed to seek professional help, and patients from private institutions not considered in the official figures. Recent estimates of excess deaths worldwide from 2020 to 2021 report that Mexico may have had 325.1 deaths per 100,000 people (Latin America and the Caribbean were estimated at 254.0, whereas the worldwide excess deaths are calculated at 120.3, in contrast) (47).

**FIG 2:**
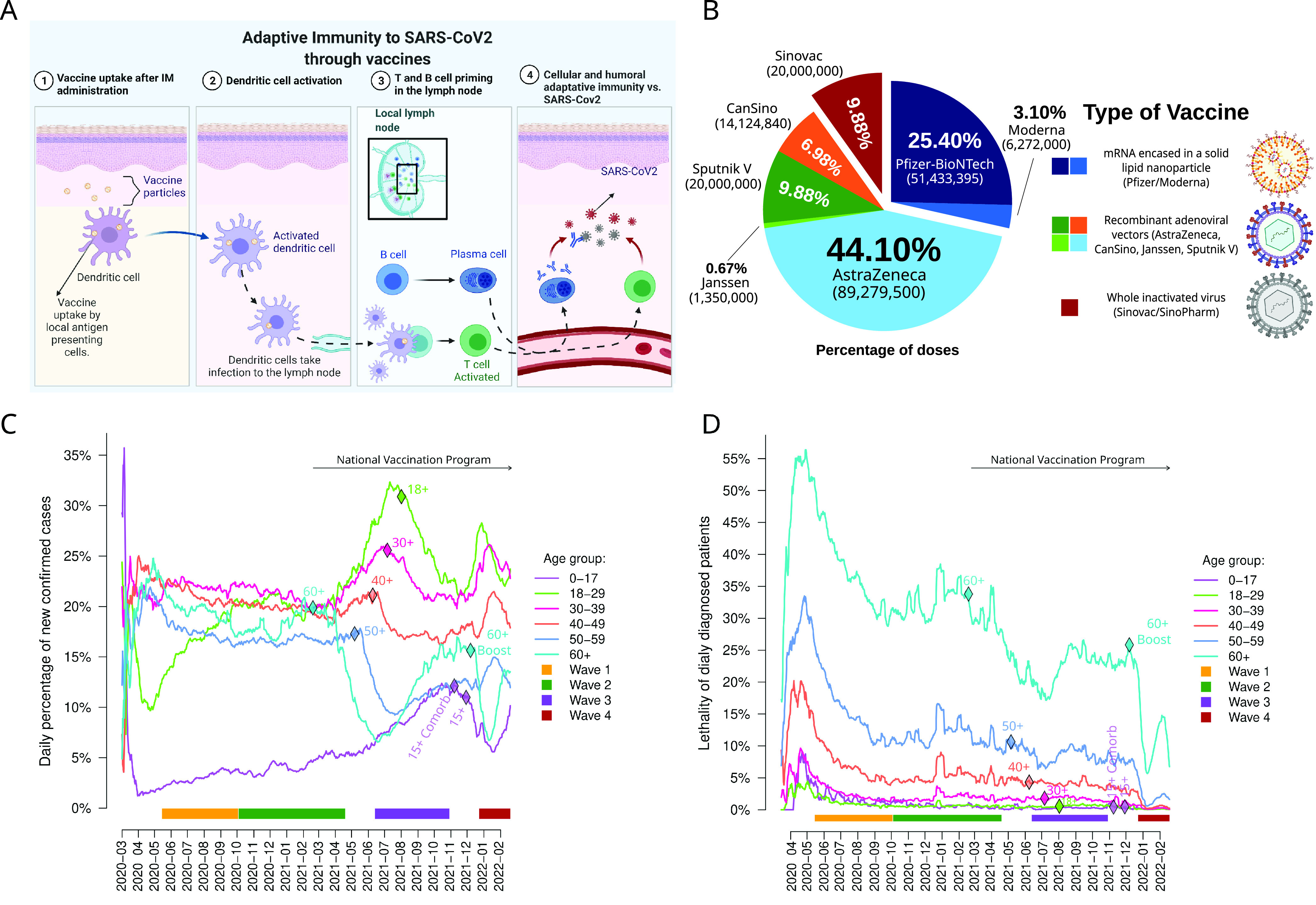
Vaccination in Mexico. (A) Mechanisms of the adaptive immunity after vaccination for controlling SARS-CoV-2 infection. (B) Total doses and percentage of each vaccine that has been acquired in Mexico. These are clustered by type of vaccine, along with example particles shown on the right. (C) Daily percentage of confirmed cases in each age group based on vaccination program (based on 7-day rolling averages). The first day of each vaccination stage (per age group) is marked with a diamond. The span of each of the fourth waves is shown as bars at the bottom. (D) Daily lethality per age group (based on 7-day rolling averages). Confirmed cases and deaths were sorted by the same date of the registry. The first day of each vaccination stage (per age group) is marked with a diamond.

Out of the 5,508,629 diagnosed patients reported in Mexico, 4,841,562 (87.89%) did not require hospitalization and had an associated 0.44% lethality, whereas 667,067 (12.11%) have been hospitalized, with a lethality of 40.50%. Of the latter, 82,998 have required mechanical ventilation (1.51% of all diagnosed patients, 12.44% of the hospitalized patients), and thus the associated lethality is higher, 83.68%. Age remains the major determinant of the COVID-19 outcome for patients worldwide, which is observed in Mexico by separating the ages and types of patients (Fig. 2B). The great majority of ambulatory patients survived (>87% in all age groups), whereas most intubated patients died (only groups between 0 and 14 have a <50% lethality). Hospitalized patients younger than 65 years of age have <50% lethality, which worsens increasingly with age (maxing out at ~60% in patients 85 years or older).

In response to the evolution of the pandemic in Mexico, the National Ministry of Health periodically adjusts nationwide guidelines (the latest Spanish version of all guidelines can be found in reference 22). The governmental epidemiological response is based on a representative sample, obtained from 475 hospitals and clinics in the USMER network (Unidad de Salud Monitora de Enfermedad Respiratoria Viral), which includes public institutions spanning the whole country (48, 49). It reports from SINAVE, a large collaborative network including public and private clinics and hospitals established for the surveillance of respiratory diseases. Nationwide weekly estimates of the total number of real cases in the entire population, collectively known as the “sentry model,” are based on these data and immunological randomized survey records, but as of February 2022, these are not publicly available. Official estimates of the early stages of the pandemic were ~25-fold the number of sampled cases (confirmed cases: 2,236, actual cases: 55,951 up to week 14, 2020 [50]).

## FOUR EPIDEMIC SURGES (WAVES)

According to the government handling of the pandemic, four nationwide epidemic surges (waves) have occurred to date. Based on a 7-day rolling daily average of confirmed cases ordered by the time patients were diagnosed, peaks occurred on 18 July (57.73 cases per million people) 2020, 10 January (126.74) and 10 August (151.66), 2021, and 15 January 2022 (462.01). Likewise, the lowest averages between surges were observed on 2 October 2020 (361.86), 13 May (125.29), and 12 December 2021 (114.29). By considering the mean of these lowest records as a basal daily average, the first wave can be delimited from 15 May 2020 to 3 October 2020, with a maximum of 0.63 daily deaths per million people on 18 July. This occurred prior to the onset of VOCs and was dominated by variants B.1 and B.1.609 and a rising B.1.1.222 variant (Fig. 1C).

New cases remained high as the second wave started, continuing from 3 October 2020 until 19 April 2021. This coincided with the winter holidays and ended after vaccination entered a nationwide phase. It has been reported that the accumulation of mutations in the variant B.1.1.222 during this wave may have led to its evolution into variant B.1.1.519, which became dominant and swiftly overtook all Mexican territories (>80% of all sequenced genomes were B.1.1.519 nationally in late March 2021). During this period, surveillance also increased in the capital, Mexico City, as antigen tests were included in the official protocols. Free testing was made widely available. In cases where a symptomatic individual was related to a SARS-CoV-2 case, laboratory confirmation was not required as it was assumed a SARS-COV-2 positive sample. The second wave also had the highest mortality of the pandemic to date, with an average of 1.13 daily deaths per million patients diagnosed on 22 January 2021. VOCs Alpha and Gamma were first detected in Mexico during the descent of this wave and eventually displaced most other variants, including B.1.1.519. This also occurred as vaccination rates increased nationwide to include younger age groups.

The evolutionary advantage of a newer VOC, Delta, allowed it to swiftly replace most other circulating variants and led to the third wave of the pandemic, spanning from 13 June to 29 October 2021. This wave had a new record of daily average new cases (19,110.14), but maximum deaths remained lower (0.63 per million people) in samples from 21 August, similar to the first wave’s maximum. As Delta overturned the rest of the variants (99% of all sequenced genomes were Delta by the end of August), its sublineages AY.20 and AY.26 became the most prevalent in the country.

Lastly, a fourth wave, which has been dominated by the Omicron VOC (mostly BA.1.1) has lasted from 23 December 2021 and is currently ongoing (still descending). The maximum daily 7-day average was an unprecedented 3.08 times the average of the third wave’s maximum, at 467.01 per million people. Average daily deaths peaked at 0.351 per million on 30 January.

## THE IMPACT OF APPLYING A WIDE RANGE OF COVID-19 VACCINES AND HERD IMMUNITY

In Mexico, seven different vaccines have been applied to the population in order to elicit the immune response against the receptor binding domain of the SARS-CoV-2 spike protein. Once administered, the vaccine particle is recognized by a local antigen-presenting cell, which in turn travels to the nearest lymph nodes and kick-starts the adaptive immune response. This turn allows for specific cytotoxic and humoral immune responses against SARS-CoV-2 epitopes (Fig. 2A) (28).

Mexico has had a slowly executed COVID-19 vaccination program compared to high-income countries, as vaccines have only been made available by the official governmental vaccination programs. These were divided by age group and started at different times, based on regional vaccine availability (the national health system is decentralized across 32 states that have partial autonomy over policy decisions) (51). Roughly, vaccination started in late December 2020 for medical staff in public hospitals and expanded to all elderly (60 and up) in February 2021. Vaccination rates ramped up throughout 2021 as new vaccines were included in the governmental programs and worldwide production increased, with groups age 50 to 59 starting in May 2021, 40 to 49 in June, 39 to 49 in July and 18 to 29 in August. In November, the 15- to 17-year-old group was added to vaccination programs, starting with those who have comorbidities. The government has justified not including younger people due to the lower lethality in the group 0 to 14 years of age. The group comprising those 60 and older was the first to receive a booster shot, starting in December 2021, and all other groups followed shortly after during January and February 2022.

As of February 2022, the Mexican government has acquired over 202 million doses and reported vaccinating 85,238,025 people, with a full primary vaccination schedule for 78,945,844 persons (62.65%) and a partial schedule for 6,292,181 (4.99%) people from all ages (population = 126,014,024).

These percentages represent most of the adult population, as the official vaccination programs have not included individuals under 15 years of age. Similar to other developing countries, vaccine rollout in Mexico was initially affected by the limited global availability, which resulted in the government acquiring multiple types of vaccine (52). Thus, the Mexican population has been immunized using seven different COVID-19 vaccines (Fig. 2B). Adenoviral vector vaccines represent the majority of vaccines used in Mexico thus far, with ChAdOx1-S (AstraZeneca) being the most used, followed by Gam-COVID-Vac (Sputnik V), Ad5-nCoV (CanSino), and Ad26.COV2-S (Janssen). Two mRNA vaccines, BNT162b2 (Pfizer-BioNTech, henceforth Pfizer) and mRNA-1273 (Moderna) represent 28.50% of the vaccines applied in Mexico and have been mainly used to immunize highly populated urban areas. To our knowledge, direct head-to-head comparisons of these vaccines have not been made as of the writing of the manuscript. Such a diverse spectrum of vaccines can have potential consequences for long-term immunity, as few vaccines have such data. As evidence continues to grow, the overall effectiveness of the vaccination program will be seen.

On this matter, a non-peer-reviewed study conducted by Bednarski et al. evaluated antibody and B cell immunity in a Mexican sample of 197 participants that received 5 different vaccines: Pfizer, AstraZeneca, Sputnik V, CanSino, and CoronaVac (Sinovac), 55 of whom had heterogeneous immunity derived from previous infection and vaccination. The previous infection could not be discarded in CoronaVac recipients, as it is a whole inactivated vaccine. When evaluating the activity of neutralizing antibodies against Omicron, neutralizing antibody titers at 50% inhibition (NT50) fell below the detection limit in all uninfected participants who had been vaccinated with Pfizer, AstraZeneca, Sputnik, CanSino, and Sinovac. As previously shown in other studies, infection increased the titers of neutralizing antibodies for SARS-CoV-2 in recipients of all vaccines applied in Mexico (53). This study is one of the few that show us a panorama of the immune responses induced by the variety of vaccines used in the COVID-19 immunization program in Mexico. It is relevant to mention that the adenoviral vector vaccines, which showed intermediate percentages of spike-specific memory B cells, represent 61.63% of vaccines used in the Mexican population. However, recent studies have shown similar T cell responses induced by different vaccine types of cross-recognized early SARS-CoV-2 variants (53, 54).

As of February 2022, measuring the actual impact that vaccination has had in Mexico is not possible since information regarding vaccination is not publicly available beyond raw totals of vaccines acquired and applied, and neither are nationwide immunological assays. However, the epidemiological data can give an insight into how each age group fared right after vaccination programs started for them (Fig. 2C and D). Since the start of the vaccination programs, most confirmed cases have been reported in younger groups (<40). However, the daily percentage of new confirmed cases saw a large decrease in newly diagnosed cases in the 60+ group following the start of the vaccination program for the elderly, which had a slow rollout due to limited vaccine availability (Fig. 2C). A similar drop was observed for each group after they were first vaccinated. Interestingly, the daily percentage of confirmed cases from the 60+ group increased after the onset of the third wave, starting about 6 months after the vaccination, which may have to do with the natural contraction and waning of the immune response. The application of booster vaccinations started during the first week of December in a sequential manner, targeting the 60+ group and then younger groups to counter waning immunity. The fourth wave, driven mostly by the dominant Omicron variant, affected groups in a stepwise manner (Fig. 2C), first affecting younger vaccinated groups, and eventually moving to increasingly older groups. In contrast, lethality per group has always been much higher in the elderly (Fig. 2D), but it has decreased with time regardless of age. Most notably, the 60+ group has seen a decrease from ~55% lethality during the first wave, to ~35% during the second one, 25% during the third one, and ~17% during the fourth one. Lethality for the other groups has remained below 15% throughout most of the pandemic, except for the first wave, and there was <5% lethality during the fourth wave.

Population immunity can be defined as the percentage of the population that is considered to be protected against SARS-CoV-2 infection due to either natural infection or vaccination and is important for studying the epidemiological trajectory and adjusting disease control strategies (55). Furthermore, there are four factors affecting population immunity: (i) vaccination with the currently available COVID-19 vaccines effectively protects from death and severe disease but does not provide sterilizing immunity (prevent effective virus infection into the host), and not all vaccines have the same effectiveness against infection or severe disease; (ii) immunity from natural infection may result in a broad spectrum of immunological memory; (iii) the emergence of variants with higher transmissibility and basic reproduction numbers (e.g., VOCs) and the ability to evade neutralizing antibodies acquired by previous vaccination or natural infections are resulting in increasing cases of breakthrough infections and reinfections; (iv) a percentage of people are not willing to receive a COVID-19 vaccine (vaccine hesitancy). These factors have contributed to the difficulty of achieving herd immunity and overall population immunity.

According to data from the National Ministry of Health of Mexico, 90% of the adult population has received at least one dose of the COVID-19 vaccine, and only 52% of teenagers from 15 to 17 years old have, as of mid-February, received at least one dose (this age group was first included in the governmental vaccination program in December 2021). Children from 5 to 14 years have not been included in any of the official vaccination programs in Mexico yet. Considering that the population from 0 to 15 years old represents 25.20% of the Mexican population (56), Mexico will have a difficult time achieving the 70% vaccination coverage target set by the World Health Organization (WHO) as its recommendation for mid-2022 if this group is not included (57). This means that Mexico has a high percentage of unvaccinated among the young population, in whom the level of immunity remains unknown, as it is difficult to calculate the role of natural infection in this population due to the low percentage of tests performed in all age groups. Age vaccination restrictions and the arrival of new variants with higher transmissibility or immune evasion capabilities might render achieving herd immunity (a high population immunity coverage that may stop the virus from spreading further; ~85%) impossible in Mexico.

Mexico is a country where social inequalities have a deep impact on health care. The COVID-19 pandemic has highlighted this issue, as rural areas are restrictive for the distribution of mRNA vaccines, while some individuals have been vaccinated abroad. According to Ortiz-Hernandez et al., municipalities with greater marginalization have a greater risk of presenting severe COVID-19, and indigenous populations are at greater risk of hospitalization and death. Additionally, the risk of death was lower for cases treated in the private and military sectors (22.74% and 28.04% of all hospitalized patients, respectively) compared to the public and university sectors (44.45% and 39.81%, respectively) (58).

The Omicron variant’s high transmissibility and immune evasiveness have resulted in a high incidence of COVID-19 cases worldwide, despite the widespread seroprevalence of neutralizing antibodies in the population. However, we still have to assess if the decoupling of cases from severe disease may be mostly due to cellular immunity from natural infection and vaccination or to its reduced virulence and its tendency to infect upper airways preferentially (59).

## CONCLUSION/PERSPECTIVES: HOW TO PREPARE FOR A FUTURE PANDEMIC?

In several countries, the rapid spread of SARS-CoV-2 emerging VOCs (such as Delta and Omicron) remained underestimated until the number of hospitalizations reached a critical saturation level. This has led the scientific community to highlight the importance of genomic surveillance of new viral variants as a tool for controlling transmission. The genomic surveillance model that was adopted, despite lower financial resources (compared to more developed countries), led to a successful program that is among the 20 countries producing the most sequences worldwide and second in Latin America, just after Brazil (30). This resulted in the identification and characterization of important local transmission clusters of variants B.122 and B.1.1.519. The identification of potential VOIs and VOCs in Mexico allowed for a better government response, as well as optimizing the health care resources.

The Organisation for Economic Co-operation and Development (OECD) recognizes Mexico as a middle-income country and among the 20 wealthier countries in the world. However, almost half of Mexico’s population is below the poverty line (43.9% as of 2020) (60). In Mexico, socioeconomic inequality prevents the large majority of workers from having any form of unemployment insurance or alternative incomes that may allow them to work remotely or suspend activities during epidemiologic surges (61). The COVID-19 pandemic has been a challenge for all health care systems and, in Mexico, it has made evident a long-overdue reform of a fractured health care system. The Mexican health care system was previously overloaded, and the COVID-19 pandemic worsened the situation (62). In the future, preventive medicine in Mexico must target specific diseases such as type 2 diabetes mellitus, systemic arterial hypertension, and chronic kidney disease, all of which are highly prevalent and are risk factors for severe COVID-19 (5, 63).

The high prevalence of such conditions is one of the reasons why containment and confinement policies have been reported to be less effective with respect to mortality and morbidity. This is particularly true for lower-income populations, where inequality is further highlighted by restricted access to health services, such as in rural areas in Mexico (64). Novel mRNA vaccines used in Mexico (Moderna and Pfizer) have stringent temperature and storage requirements that prevent them from being distributed in rural and low-income regions (65, 66). In contrast, viral vector vaccines such as AstraZeneca, having more lenient storage conditions (67), were more widely distributed in these areas. Like in other countries, Mexico followed a vaccination program that prioritized patients with risk factors, such as the elderly or the highly exposed, such as teachers and health care professionals.

Like many Latin American countries, the disjointed health care system in Mexico, misinformation, and lack of authorities’ leadership to apply global health care measures (e.g., during early stages of the pandemic, wearing masks was not officially endorsed by the federal government but was instead dependent on local authorities) are factors that contributed to the current situation, even though decision-making is mostly based on the WHO and the Pan American Health Organization guidelines. Although the vaccine supply in Mexico has been better than in other Latin American countries, the Mexican vaccine rollout has been slow and uneven (51). Due to its slow pace, it has failed to meet its original goal of completing 18+ vaccination (summer 2021) and for failing to include the youngest in the population. Facing an expected limited worldwide vaccine supply and aggravated by its imbalanced distribution (68), the Mexican government invested in the development of a Newcastle vector vaccine that was to be rolled out and produced locally by late 2021; however, as of the writing of this article, this is still undergoing phase II trials (51).

The fragmentation of the Mexican health care system was deeply exposed by the COVID-19 pandemic, as Mexico has had one of the highest numbers of infections and deaths among health care professionals (51), accounting for over 4,628 confirmed and 113 unconfirmed 1.59% (+0.04%) COVID-related deaths among 290,769 confirmed cases in the last official report (18 December 2021 [69]). Stress and inadequate working conditions, as well as underlying chronic diseases, have been proposed to be additional risk factors contributing to this situation (70). Limited SARS-CoV2 testing and lack of personal protective equipment were shown to be points of improvement. Such shortcomings must be addressed, as they would benefit the entirety of the Mexican population (71).

In order for Mexico, and for other countries in similar scenarios, to overcome the current COVID-19 pandemic and meet the goal set by the WHO (70% vaccination by mid-2022 [72]), vaccine rollout will need to take all ages into account, including children under 15 years of age. Currently, Mexico has started the vaccination of children between 12 and 17 (http://vacunacovid.gob.mx/wordpress/vacuna-covid19-adolescentes/) and recently launched the registration process for vaccination of children between 5 and 11 years (http://vacunacovid.gob.mx/wordpress/vacunacion-contra-covid-19-para-ninas-y-ninos-de-5-a-11-anos/). However, vaccination for younger children (between 5 years and 6 months) will need the support of clinical trials before their immunization can be performed. For instance, the Moderna vaccine phase 2/3 placebo-controlled expansion study in children 6 months to less than 12 years of age is under way (73). As with other diseases, universal vaccination programs are key and are a gateway to end the COVID-19 pandemic in the long term.
